# BioNano genome mapping of individual chromosomes supports physical mapping and sequence assembly in complex plant genomes

**DOI:** 10.1111/pbi.12513

**Published:** 2016-01-23

**Authors:** Helena Staňková, Alex R. Hastie, Saki Chan, Jan Vrána, Zuzana Tulpová, Marie Kubaláková, Paul Visendi, Satomi Hayashi, Mingcheng Luo, Jacqueline Batley, David Edwards, Jaroslav Doležel, Hana Šimková

**Affiliations:** ^1^ Institute of Experimental BotanyCentre of the Region Haná for Biotechnological and Agricultural ResearchOlomoucCzech Republic; ^2^ BioNano GenomicsSan DiegoCAUSA; ^3^ Australian Centre for Plant Functional GenomicsUniversity of QueenslandBrisbaneQLDAustralia; ^4^ School of Agriculture and Food SciencesUniversity of QueenslandBrisbaneQLDAustralia; ^5^ Department of Plant SciencesUniversity of CaliforniaDavisCAUSA; ^6^ School of Plant BiologyUniversity of Western AustraliaCrawleyWAAustralia

**Keywords:** optical mapping, wheat, sequencing, physical map, flow sorting, chromosomes

## Abstract

The assembly of a reference genome sequence of bread wheat is challenging due to its specific features such as the genome size of 17 Gbp, polyploid nature and prevalence of repetitive sequences. BAC‐by‐BAC sequencing based on chromosomal physical maps, adopted by the International Wheat Genome Sequencing Consortium as the key strategy, reduces problems caused by the genome complexity and polyploidy, but the repeat content still hampers the sequence assembly. Availability of a high‐resolution genomic map to guide sequence scaffolding and validate physical map and sequence assemblies would be highly beneficial to obtaining an accurate and complete genome sequence. Here, we chose the short arm of chromosome 7D (7DS) as a model to demonstrate for the first time that it is possible to couple chromosome flow sorting with genome mapping in nanochannel arrays and create a *de novo* genome map of a wheat chromosome. We constructed a high‐resolution chromosome map composed of 371 contigs with an N50 of 1.3 Mb. Long DNA molecules achieved by our approach facilitated chromosome‐scale analysis of repetitive sequences and revealed a ~800‐kb array of tandem repeats intractable to current DNA sequencing technologies. Anchoring 7DS sequence assemblies obtained by clone‐by‐clone sequencing to the 7DS genome map provided a valuable tool to improve the BAC‐contig physical map and validate sequence assembly on a chromosome‐arm scale. Our results indicate that creating genome maps for the whole wheat genome in a chromosome‐by‐chromosome manner is feasible and that they will be an affordable tool to support the production of improved pseudomolecules.

## Introduction

Recent progress in understanding eukaryotic genome structure and function lead to the realization that a majority of genome sequences is transcribed and that, in addition to protein coding sequences, the so‐called noncoding DNA may also be functionally significant (ENCODE Project Consortium, 2012). In addition, unexpected plasticity of eukaryotic genomes, and functional significance of copy number and structural variation, has been revealed (Zarrei *et al*., 2015). These observations underline the need for high‐quality reference genome sequences, which are a prerequisite to study these phenomena and discover genome features other than genes underlying traits of agronomic importance. While next generation sequencing (NGS) technologies excel in huge throughput, reaching as much as trillions base pairs within a few days, the prevalent technologies provide short reads of only several hundred base pairs, making the assembly of large and complex genomes a daunting task.

As discussed recently, the published reference genome sequences obtained using whole‐genome shotgun strategies may suffer from extensive mis‐assemblies and comprise gaps (Ganapathy *et al*., 2014; Pendleton *et al*., 2015; Ruperao *et al*., 2014). This is also true to some extent for genome assemblies obtained even using the robust BAC‐by‐BAC approach (Callaway, 2014; Shearer *et al*., 2014), indicating problems with the assembly of BAC‐contig physical maps. Thus, not a single plant or animal genome is truly complete (Kelley and Salzberg, 2010), and even the golden standard sequence of the human genome is known to be missing some genomic regions (Callaway, 2014). Wider application of technologies providing reads in the kilobase range, such as single molecule real‐time sequencing adopted by Pacific Biosciences (Chaisson *et al*., 2015), and nanopore technologies (Mikheyev and Tin, 2014) promise to improve the quality of whole‐genome shotgun assemblies. Yet, even reads of this length are not enough, as it has been estimated that reads exceeding 200 kb would be needed to resolve repeats and other problematic regions (Marx, 2013). Current NGS technologies fall short of this, and thus, the introduction of errors in whole‐genome assemblies cannot be avoided, which may negatively influence various downstream applications.

Several approaches have been applied for validation and correction of genome assemblies. For example, errors in clone‐based physical maps and pseudomolecule mis‐assemblies can be identified using fluorescence *in situ* hybridization (FISH) with BAC clones as probes. Using this method, Shearer *et al*. (2014) showed that scaffolds representing one‐third of the tomato genome were arranged incorrectly. Unfortunately, BAC‐FISH is time‐consuming and laborious, and its applicability in large genomes is hampered by the presence of dispersed repetitive DNA, which make preparation of single‐copy probes a tedious task (Janda *et al*., 2006). Using a recently developed approach, incorrect assignment of sequences to particular chromosomes can be revealed by sequencing DNA of flow‐sorted chromosomes (Ruperao *et al*., 2014). Although this approach does not detect errors in ordering clone or sequence contigs within a chromosome, its relative simplicity makes it applicable in all species, from which chromosomes can be sorted. The principles of optical mapping were developed some time ago (Zhou and Schwartz, 2004), but only recently the technology and its modifications, such as genome mapping in nanochannel arrays (Lam *et al*., 2012), became suitable for mapping large genomes (Dong *et al*., 2013; Ganapathy *et al*., 2014; Hastie *et al*., 2013; Pendleton *et al*., 2015; Shearer *et al*., 2014; Young *et al*., 2011; Zhang *et al*., 2015; Zhou *et al*., 2009). The method produces physical maps of short sequence motifs (i.e. recognition sites of nicking/restriction enzymes) along hundreds to thousands of kilobase‐long stretches of DNA, and provides a high‐throughput tool for ordering and orienting contigs of physical maps and validation of genome assemblies.

Bread wheat (*Triticum aestivum* L.), together with rice (*Oryza sativa*, L.) and maize (*Zea mays* L.), are the three most important crops and significant sources of calories and proteins for humankind. However, their genomes differ considerably in size and complexity, with bread wheat having by far the largest (~17 Gb) and polyploid genome consisting of three homoeologous subgenomes, A, B and D, with inter‐ and intrachromosomal duplications, and a high proportion of repetitive DNA (e.g. 85% for chromosome 3B; Choulet *et al*., 2014a). The availability of a wheat reference genome sequence is needed urgently to employ molecular and genomic tools more extensively to speed up breeding improved varieties (Choulet *et al*., 2014b; Feuillet *et al*., 2012). The availability of a genome sequence would also make wheat an attractive model to study genome changes accompanying evolution of polyploid crop genomes and their domestication. While various strategies have been employed to tackle the huge and complex bread wheat genome, including shotgun sequencing of the whole‐genome (Brenchley *et al*., 2012; Chapman *et al*., 2015) and shotgun sequencing of chromosomes isolated by flow sorting (IWGSC, 2014), it became obvious that a high‐quality genome sequence cannot be obtained from short‐read shotgun data.

Considering the peculiarities of the wheat genome, The International Wheat Genome Sequencing Consortium (IWGSC) selected a clone‐by‐clone sequencing strategy based on physical maps constructed from chromosome (arm)‐specific BAC libraries as a key approach towards obtaining the reference genome sequence (http://www.wheatgenome.org/). This approach offers a lossless reduction in complexity, in which the genome is sequenced *per partes*; avoids problems due to genome size, polyploidy and large duplications; and greatly simplifies the genome assembly. To completely reconstruct the genomic sequence from BAC sequence data, contigs of the physical map are anchored and oriented. This relies on markers that are present in the contigs, and whose position on chromosomes is known. To satisfy this demand, large numbers of markers evenly distributed along chromosomes are needed. While high‐density linkage maps of wheat were recently constructed using high‐throughput approaches, their resolution is limited due to relatively small sizes of the mapping populations. A particular challenge is posed by low‐recombining regions, which may represent more than one‐third of the chromosome, and in which the resolution of genetic maps is poor (Erayman *et al*., 2004; Luo *et al*., 2013; Paux *et al*., 2008). Radiation hybrid (RH) maps (Kumar *et al*., 2012; Tiwari *et al*., 2012) are largely independent of recombination and may aid in resolving this problem, but are not yet available for each of the wheat chromosomes. Alternative recombination‐independent approaches are thus needed, and the BioNano genome mapping appears highly promising in this respect.

High accuracy of the genome mapping in nanochannel arrays enables *de novo* assembly of genome maps even without prior knowledge of genome sequence (Lam *et al*., 2012). However, the huge and polyploid bread wheat genome appears too complex to be analysed as a whole. Moreover, as the reference genome sequence is being produced by sequencing physical maps of individual chromosomes, or chromosome arms, it seems practical to follow the chromosome‐based strategy of IWGSC and produce BioNano maps from individual chromosomes. Here, we chose the short arm of chromosome 7D (7DS) with the size of 381 Mb (Gill *et al*., 1991; Šafář *et al*., 2010) as a model to demonstrate for the first time that it is possible to couple chromosome flow sorting with genome mapping in nanochannel arrays to create a *de novo* genome map. DNA prepared from flow‐sorted chromosomes was of superior quality and enabled construction of a high‐resolution chromosome map. Moreover, long molecules achieved by our approach facilitated chromosome‐scale analysis of repetitive sequences. Anchoring the 7DS genome map to the 7DS sequence assemblies obtained by clone‐by‐clone sequencing provided a valuable tool to improve the physical map and validate sequence assembly of the chromosome arm.

## Results

### *De novo* assembly of a 7DS genome map

The genome map of the 7DS chromosome arm of wheat was built from molecules treated by the nicking enzyme *Nt.BspQI* (labelled motif GCTCTTC). Statistics for data collection and genome map assembly are given in Table 1. In total, 68.8 Gb data of DNA molecules over 150 kb were collected from one Irys chip, which corresponds to 180 equivalents of the 7DS chromosome arm. This coverage was compiled of 209 788 molecules, the largest of which exceeded 2 Mb in size (Figure S1). The N50 of the size‐filtered molecules (>150 kb) was 354 kb. The 7DS genome map was assembled *de novo* and consisted of 371 constituent genome maps with average length of 0.9 Mb and N50 of 1.3 Mb. The size of the largest genome map was 4.6 Mb. The 7DS genome map has a total length of 350 Mb and covers 92% of the estimated arm length.

**Table 1:** Data collection and assembly statistics

	No. molecules/genome maps	Total length	7DS arm coverage	Molecule/map N50	Longest molecule/map (Mb)
Single molecules (>150 kb)	209 788	68.8 Gb	180×	354 kb	2.1
Map assembly	371	350 Mb	0.92×	1.3 Mb	4.6

### Tandem repeat detection and analysis

**Figure 1:**
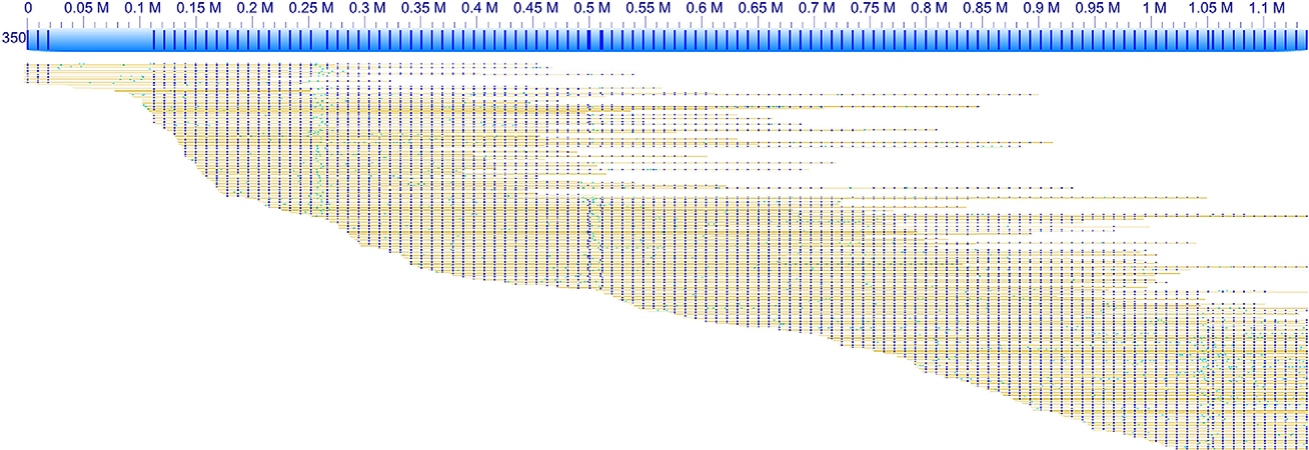
Genome map No. 350 comprising a long array of tandem repeats. The pile of single molecules, depicted as yellow lines with blue and green dots corresponding to mapped and unmapped labels, respectively, was a source for building the consensus genome map (blue bar). The regular labelling pattern indicates presence of tandem repeats.

Long DNA molecules obtained in our study enabled chromosome‐scale analysis of repetitive sequences. During image acquisition on Irys, striking DNA molecules can be seen that have evenly spaced labels (i.e. fluorescently labelled *Nt.BspQI*‐nicked sites) that span over hundreds of kilobases. The regular labelling pattern indicates the presence of tandem repeats. In the wheat 7DS data, a particular labelled segment was seen with 9.3 kb spacing that spreads over a region of ~1 Mb in the genome map No. 350 (Figure 1). Among single molecules underlying this map, we detected several comprising arrays of the evenly spaced labels of a minimum of 800 kb in length. Anchoring the available 7DS sequence scaffolds to the genome map No. 350 did not provide any significant match. This indicates the genome mapping revealed a hitherto unknown genome region composed of a long tandemly organized repeat, which is in its entirety intractable by traditional sequencing methods. Quantitative analysis of labelled tandem repeats within the whole 7DS dataset revealed that the majority of these repeats fall into the size category of 9.25–9.75 kb (Figure S2), to which significantly contributes the repeat constituting the map No. 350. Potentially, the peak in repeat size can represent one type of repeat only and the size span is given by mutations or by variability in stretching among single molecules.

### Optimization of sequence anchoring

The genome map can serve as a guide for sequence assembling, provided available sequence contigs/scaffolds are long enough to be reliably anchored to the genome map. To determine the minimal sequence length needed for reliable anchoring within a wheat chromosome arm, we randomly selected ten BAC clones with inserts over 120 kb and typical labelling frequency (~12 sites/100 kb) assembled as one contiguous sequence (Table S1). Comparison of these clones with the complete set of genome maps revealed their locations, which were determined as the best hits, reaching confidence value ranging from 15.85 to 24.89. The allocations were confirmed through anchoring of overlapping or neighbouring clones, which in all cases hit the selected genome map. Identical position for each of the clones was also obtained after truncating them to the size of 120 kb. Using a sliding window approach, 210 sequence fragments of three size categories (30, 60 and 90 kb) were generated. These comprised 100, 70 and 40 sequences of 30, 60 and 90 kb, respectively. Applying this approach, a variety of nicking site patterns were obtained for each size category.

Comparison of the 210 sequences with genome maps provided multiple hits for all of the sequences. The best hit (highest confidence value) in the correct position was observed for 109 of them (52%). Data for particular size categories are given in Table 2. From the total number of one hundred 30‐kb sequences, only 12% were assigned to the correct position, though with generally low confidence values (5.86–7.60). In the 60‐kb size category, 57 of 70 (81%) sequences were assigned correctly with confidence ranging from 6.07 to 13.87. The most reliable anchoring results were obtained with 90‐kb sequences. In this category, all 40 sequences gained the highest confidence value for the correct genome‐map position. The variation in confidence level within a size category was mainly due to differing number of recognition sites of the nicking enzyme (Table 2): higher density of recognition sites generally increases reliability of the assignment.

**Table 2:** Assignment of 30‐, 60‐, and 90‐kb sequences to 7DS genome maps

Sequence length (kb)	No. sequences	No. correctly assigned	Percentage correctly assigned (%)	Lowest confidence Highest confidence	No. labels^a^
30	100	12	12	5.86	5
				7.60	6
60	70	57	81	6.07	5
				13.87	10
90	40	40	100	7.91	6
				19.99	14

a No. labels corresponds to number of distinguishable *Nt.BspQI* recognition sites in the sequence.

The study indicated that without additional information, 30‐kb sequences could not be reliably assigned to a genome map. In the 60‐kb category, 70% sequences could be anchored with confidence value above 7. Knowledge of the sequence context, for example other sequence contigs belonging to the same or a neighbouring BAC clone known from the physical map, can aid reliable assignment of the short contigs through co‐anchoring of the short sequences to genome maps. With preceding determination of a corresponding genome map, nearly double of the 30‐kb sequences (21%) could be assigned to the right position. For 60‐kb sequences, the percentage of correctly positioned sequences rose from 81% to 87%. This approach can be used to order and orientate shorter sequence contigs within a BAC clone or a pool of overlapping BAC clones.

Comparison of genome maps with complete sequences of the above BAC clones (in total 1376 kb sequence) enabled investigating error in size measurement introduced by mapping in nanochannel arrays. The size estimates showed to be highly precise, underestimating the sequence length by 1.4 kb (±0.56 kb) per 100 kb sequence.

### BAC‐contig scaffolding and validation

**Figure 2:**
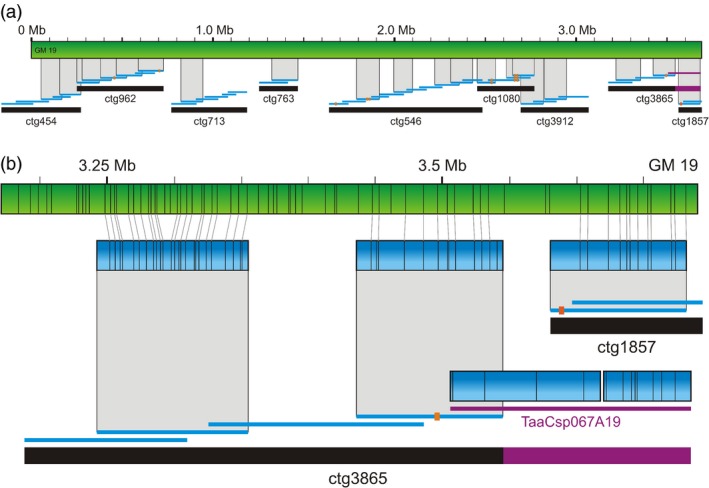
Scaffolding and correcting physical map contigs based on the genome map No. 19. (a) In total, nine contigs of the physical map (black bars) could be anchored through sequences of constituting BAC clones (blue lines) to the genome map No. 19 (green bar). Short red bars indicate approximate positions of *Aegilops tauschii* SNP markers anchored to particular clones. The purple line and bar in ctg3865 represent clone TaaCsp067A19, which was incorrectly assigned to this contig. Detail is shown in (b). A *cmap* of the clone TaaCsp067A19 does not match the corresponding region in GM19. The green bar corresponds to GM19, while the blue bars represent *in silico* digested BAC sequences (*cmaps*).

Long genome maps spanning several BAC contigs serve as a guide for building contig scaffolds in the length of megabases with precisely estimated gap sizes. They can also point to potential contig overlaps and mis‐assemblies, as demonstrated in Figure 2 for genome map No. 19 (GM19). This map with a length of 3.69 Mb is one of the largest in the assembly, thus having a potential to span several contigs of the physical map. Available sequence contigs of the minimum tilling path (MTP) BAC clones larger than 20 kb were aligned to GM19. Of the complete set of 5847 contigs, 21 mapped to GM19 with a significant level of confidence. These sequence contigs anchored in total nine 7DS BAC contigs covering as a whole 89% of GM19 and oriented six of them (Figure 2a). A list of MTP BAC clones from the anchored contigs is given in Table S2. Contigs 713, 763 and 1857, which were anchored through one BAC clone each only, were oriented by allocating BAC‐end sequences of overlapping clones within the sequence of the anchored one. We also identified three potential overlaps between BAC contigs ctg454 and ctg962, ctg546 and ctg1080, and ctg1080 and ctg3912, respectively. The overlaps between ctg546 and ctg1080, and ctg1080 and ctg3912, respectively, were confirmed by BLAST alignment of sequences of overlapping BAC clones, while the overlap between ctg454 and ctg962 could not be validated due to the lack of sequence data. We revealed five gaps between the contigs of the physical map, covering 410 kb total. Potentially, some of these gaps can be closed in the future once complete sequence information of all MTP clones is available. In other genome maps, we confirmed that the map resolution was sufficient to resolve BAC contigs as short as three BAC clones represented by two MTP clones only. An example of a three‐clone contig successfully anchored to a genome map is ctg1974 in Figure 4.

Alignment of BAC clone sequences to the GM19 pointed to a BAC clone that was incorrectly assigned to ctg3865 (Figure 2b). In contrast to another two BAC clones of this contig, which matched GM 19 with a high confidence, the end clone TaaCsp067A19 had no match with this genome map. Sequences of the mis‐assigned clone showed no homology with sequences of overlapping clones, neither from ctg3865, nor from the potentially overlapping ctg1857. At the same time, the TaaCsp067A19 clone matched genome map No. 36, which proposed its positioning in ctg40. The proposed position has been confirmed by sequence overlaps with neighbouring clones.

### Anchoring of BAC contigs to the 7DS arm

Traditionally, BAC contigs have been ordered along chromosomes through harboured markers with known positions in genetic or RH maps, which created a big demand on the number of markers applied. A combination of various types of genomic resources (genetic and RH maps or synteny‐based tools), which need to be integrated, is typical for the majority of physical mapping projects. The BioNano genome map provides an alternative means for positioning BAC contigs and at the same time enables a straightforward integration of various maps.

GM19 positioned four BAC contigs, (ctg454, ctg713, ctg763 and ctg3912) without any marker into the context of five contigs (ctg962, ctg546, ctg1080, ctg3865 and ctg1857) that had been anchored by a total of ten markers to the *Aegilops tauschii* genetic map (Figure 2a). The marker order proposed by the genome map (Table S2) was in agreement with the order of the markers in the genetic map (Luo *et al*., 2013), which provides a support for the correctness of the genome map.

**Figure 3:**
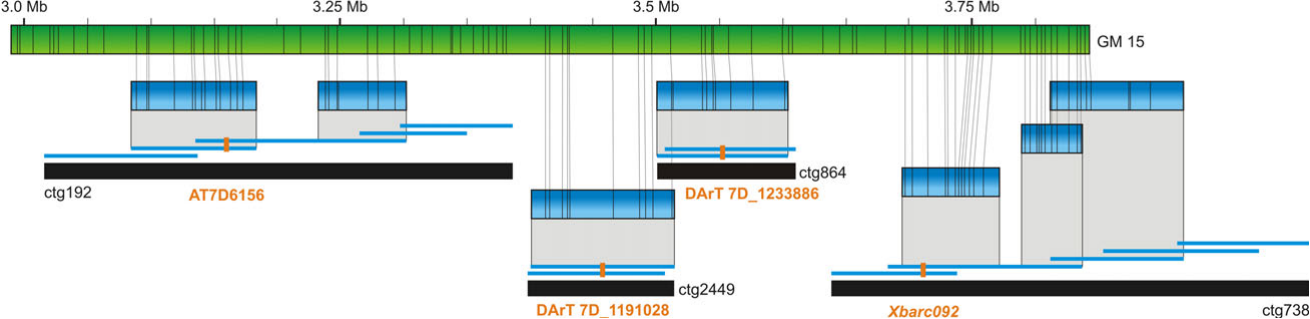
Local integration of three genetic maps through the genome map No. 15. Contigs of the physical map (black bars) were aligned to the genome map No. 15 (green bar) through sequences of constituting BAC clones (blue bars). The BAC contigs carry genetic markers (red) originating from three genetic maps, which could be integrated through the genome map.

An example of the integration of various genetic maps through a genome map is given in Figure 3. Available sequence contigs of the MTP BAC clones >20 kb were aligned to the genome map 15, which resulted in anchoring seven sequences coming from four different BAC contigs. These contigs were previously allocated by markers to a total of three genetic maps: contig ctg192 was anchored to the genetic map of *Ae. tauschii* (Luo *et al*., 2013), contigs ctg2449 and ctg864 were anchored to the consensus DArTseq map of bread wheat (A. Kilian, unpublished), and contig ctg738 was anchored to the wheat composite microsatellite map (http://wheat.pw.usda.gov/GG2/index.shtml). While the mutual positions of ctg2449 and ctg864 could be deduced from the DArTseq map and could also be confirmed by a sequence overlap between clones constituting the two contigs, the positions of ctg192 and ctg738 were only revealed from the genome map, which enabled estimating mutual positions of all the contigs as well as positions and approximate physical distances of *Ae. tauschii* SNP marker AT7D6156, DArTseq markers 7D_1191028 and 7D_1233886, and a microsatellite marker *Xbarc092*, respectively.

### Merging and scaffolding of genome maps

**Figure 4:**
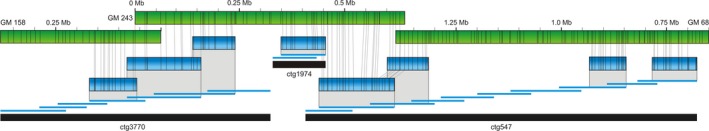
Merging genome maps. Three genome maps (green bars) could be merged together after aligning sequences of BAC clones (blue bars) from three contigs of the physical map (black bars).

Limitations for BioNano genome map assembly are posed by regions with low density of nicking sites and also ‘fragile sites’, caused by the occurrence of proximally located nicking sites on opposite DNA strands, which induce a biased fragmentation of the DNA (Lam *et al*., 2012). Both limitations can be overcome by aligning the genome maps with BAC contigs that may span the problematic region and reliably scaffold the genome maps or serve as a guide for merging particular genome maps as demonstrated in Figure 4.

Alignment of the available set of 7DS MTP sequences to genome map No. 243 yielded four reliably anchored sequence contigs, which belonged to three BAC contigs: ctg3770, ctg1974 and ctg547. As the outer contigs ctg3770 and ctg547 extended far beyond GM243, we aligned available sequences of other BAC clones from these contigs to the complete set of genome maps, which anchored genome map No. 158 proximal and genome map No. 68 distal of GM243, respectively. The alignment also revealed a small overlap between GM243 and GM158 and between GM243 and GM68, respectively. Thus, three genome maps could be joined based on the information from the physical contig map. This approach provides a potential for a significant improvement of genome map assembly parameters.

## Discussion

The principles of optical mapping were developed some time ago (Zhou and Schwartz, 2004), but only recently the technology and its modifications such as genome mapping in nanochannel arrays (Lam *et al*., 2012) became suitable for mapping large genomes. The largest optical/genome map assembled so far is that of human (3.2 Gb; Teague *et al*., 2010; Pendleton *et al*., 2015). It is obvious that such a tool is also extremely necessary for crops with larger genomes, including that of bread wheat.

As the huge size (~17 Gb) and polyploid nature of the bread wheat genome may pose a serious obstacle to assembling a map on a whole‐genome level, we proposed coupling the genome mapping with flow sorting of particular chromosomes/arms, which dissects the wheat genome into manageable portions of 224–993 Mb (Šafář *et al*., 2010). The present work on the 7DS chromosome arm demonstrates that DNA prepared from flow‐sorted chromosomes is of superior quality and allows *de novo* assembly of a quality genome map. This result confirms that the protocol of high molecular weight (HMW) DNA preparation from flow‐sorted chromosomes (Šimková *et al*., 2003), developed and applied previously for construction of chromosomal BAC libraries (Šafář *et al*., 2010; http://olomouc.ueb.cas.cz/genomic-resources), is highly compatible with the BioNano Genomics Irys platform. As the procedure of chromosome sorting has been elaborated for use in more than twenty plant species (Doležel *et al*., 2014), this approach can find a wider application. Flow sorting of particular chromosome types, which substantially reduces sample complexity and helps to deal with polyploidy and segmental duplications, is limited to chromosomes with distinct size or to the availability of special cytogenetic stocks (addition, translocation, ditelosomic lines), which enable discrimination and flow sorting of desired chromosomes (Doležel *et al*., 2014). If discrimination of individual chromosomes is not possible, fractions enriched for particular chromosomes can be sorted to reduce sample complexity (Vrána *et al*., 2015). In species, in which the chromosome sorting is not feasible, our flow cytometry‐based protocol can be used for purification of cell nuclei. In contrast to traditional methods for HMW DNA preparation, DNA prepared from flow‐sorted nuclei is not contaminated by plastid and mitochondrial DNA and the protocol greatly reduces negative effects of secondary metabolites (Šafář *et al*., 2004; Šimková *et al*., 2003).

The high quality of the HMW DNA prepared from the flow‐sorted 7DS arm, which was reflected by the large size of single molecules (Figure S1), enabled revealing an array of tandem repeats exceeding ~800 kb in length under the support of single molecules carrying a regular labelling pattern along DNA stretches of this size. Such regions are intractable to current short‐read sequencing technologies, whose output assemblies are heavily biased against repeats and duplications because of short‐read mapping ambiguity and assembly collapse (Alkan *et al*., 2011). Even long‐read technologies such as single molecule real‐time sequencing using the PacBio platform or long‐insert mate‐pair sequencing are not able to reliably span a repeat region of this size. In the light of this, the missing match in the available 7DS sequence assemblies for the genome map carrying the array is not surprising. Alternatively, the region may have been absent in the 7DS BAC library, which was the source of the sequence data, as tandem repeat regions inserted in a BAC vector induce recombination within the clone and thus are refractory to cloning. Genome mapping in nanochannel arrays relying on the analysis of single molecules of hundreds to thousands of kilobases in length proved useful for identifying regions of tandem repeats also in other organisms. Hastie *et al*. (2013) found two blocks of tandem repeats in a partial genome map of *Aegilops tauschii*, which were missing in the sequence assembly of the 2.1‐Mb prolamin gene family region. Cao *et al*. (2014) analysed structural variation in a human genome and found an intact molecule of 633 kb harbouring two tracts of 2.5‐kb tandem repeats: one of at least 53 copies; the other of at least 21 copies. In their study, the 2.5 kb showed the most abundant size category among labelled repeats in YH cell line (male), in contrast to line NA12878 (female), in which the frequency of the 2.5 kb repeat was 19 times lower. Based on additional genome mapping in other males and females, the 2.5 kb repeat appeared male‐specific, indicating a potential biological role of tandem repeats in the genome and predicting them a source of structural variability.

Besides being an invaluable tool to study structural variation, the optical/genome maps were also highly beneficial in assembling genome sequences by aiding ordering, orienting and joining contigs and scaffolds; sizing and closing gaps; anchoring the scaffolds; and identifying and correcting mis‐assemblies (Dong *et al*., 2013; Ganapathy *et al*., 2014; Hastie *et al*., 2013; Pendleton *et al*., 2015; Shearer *et al*., 2014; Young *et al*., 2011; Zhang *et al*., 2015; Zhou *et al*., 2007, 2009). The majority of current sequencing projects rely on assembling short‐read data obtained by shotgun sequencing, which results in heavily fragmented and frequently incorrect assemblies. While these can be improved through the genome maps, the length of assembled contigs/scaffolds must be sufficient to ensure reliable anchoring. Our study carried out on a wheat chromosome arm of relatively low complexity (381 Mb), but with a high proportion of repeats (over 80%), showed that sequence contigs of 90 kb could be unambiguously anchored to the 7DS genome map. We can extrapolate that the required sequence length will increase with the genome complexity and in genomes with a lower frequency of nicking sites. In our experiment, we were partially successful even with shorter sequence contigs, observing 81% and 12% of correct assignments for 60‐ and 30‐kb sequences, respectively. While the confidence value of ~6, obtained for some of the shorter sequences (Table 2), would be prohibitively low if anchoring sequences from shotgun assemblies, it can still be applied in case of the BAC‐by‐BAC approach, thanks to the support of other clones from the same BAC contig, which reliably preselect the corresponding genome map. BAC‐by‐BAC sequencing of complex crops genomes, including wheat, is frequently carried out on pools of several overlapping BAC clones (Choulet *et al*., 2014a; http://www.wheatgenome.org/). If validating, scaffolding and correcting sequence assemblies within a narrow genome region determined as a BAC contig, the alignment can be performed within one or two genome maps only, which allows decreasing the confidence value and mapping even the short sequences. This indicates that coupling the genome mapping with the BAC‐by‐BAC sequencing strategy is a powerful approach to resolving complex genomes.

With 90‐kb anchorable sequence length, the genome mapping in nanochannel arrays outperforms the optical mapping technology of OpGen, Inc., which generally requires scaffolds ≥200 kb with <5% Ns for reliable map assignment (Zhang *et al*., 2015). The reason for the difference may lie in inherent features of the two technologies. While genome mapping on the Irys platform is based on labelling DNA molecules in enzyme‐specific nicking sites and subsequent automatic massively parallel imaging of DNA molecules in nanochannel arrays (Lam *et al*., 2012), optical mapping is based on stretching long DNA molecules in a microfluidic device, attachment of molecules to the surface of the device by electrostatic interactions, subsequent digestion by specific restriction endonuclease, and staining (Zhou and Schwartz, 2004). The latter technique suffers from lower uniformity in DNA stretching and has a higher error rate (Cao *et al*., 2014), which has to be compensated by a higher coverage of input data (Zhang *et al*., 2015; Zhou *et al*., 2007, 2009). In our study, we observed high concordance between size estimates based on sequencing and the genome mapping; the genome maps in all cases underestimated the sequence length, on average by 1.4%. This systematic underestimate was probably due to nondiscriminated recognition sites whose distance was under the resolution limit of the technology (1.5 kb). Despite higher error rate in the single‐molecule data, genome maps generated by the optical mapping approach appear less fragmented and have larger contigs than those generated on the Irys platform (Ganapathy *et al*., 2014; Zhou *et al*., 2007, 2009). This is mainly due to ‘fragile sites’ associated with nick sites adjacent to each other on the opposite DNA strands, which are specific to BioNano Genomics technology. Stitching these sites through contigs of the physical map, as demonstrated in our study, or through long sequence contigs, as shown by Cao *et al*. (2014) or Pendleton *et al*. (2015), can improve the assembly metrics significantly (Cao *et al*., 2014).

In our study, we collected from one Irys chip 68.8 Gb size‐filtered data, which corresponds to 180 equivalents of the 7DS chromosome arm. This exceeds the coverage of 70–80×, required for the BioNano technology (Cao *et al*., 2014; Pendleton *et al*., 2015), and suggests that one chip may provide sufficient data for two wheat chromosome arms. This implies that the whole wheat genome could be analysed chromosome‐by‐chromosome using only 21 Irys chips, which makes the analysis of a polyploid ~17 Gb genome a realistic goal.

We demonstrate that the BioNano genome map is a useful tool for ordering and orienting BAC contigs along a chromosome. This is extremely beneficial in non‐ or low‐recombining regions of the genome, in which genetic mapping fails. Studies in wheat and its relatives revealed that low recombination rate may affect more than one‐third of chromosomal length (Erayman *et al*., 2004; Luo *et al*., 2013; Paux *et al*., 2008), which suggests that the role of genome mapping may be invaluable in a significant part of the genome. Another challenge in projects based on BAC‐by‐BAC sequencing pose short contigs of a few BAC clones that are not easy to anchor and nearly impossible to orientate. For this reason, contigs shorter than five (Paux *et al*., 2008; Philippe *et al*., 2013) or even six BAC clones (Breen *et al*., 2013; Poursarebani *et al*., 2014) were excluded from wheat physical map assemblies and are not subjected to sequencing. This approach can introduce gaps in sequence assemblies. The present study demonstrates that genome maps have a sufficient resolution to position and orientate contigs consisting of as little as three BAC clones, which can contribute to higher completeness of generated genome sequences.

To conclude, using wheat chromosome arm 7DS as a model, we demonstrate the suitability of flow‐sorted chromosomes for BioNano mapping technology. This approach facilitates physical map scaffolding, validation, correction and anchoring. As such, it provides a missing tool needed to complement the extant genomics tools to deliver high‐quality reference genome sequences and analyse structural genome variation.

## Experimental procedures

### Building BioNano genome map

High molecular weight DNA was prepared from wheat 7DS chromosome arm as described in Šimková *et al*. (2003). The 7DS arm was flow‐sorted from a double ditelosomic line of wheat *Triticum aestivum* L. cv. Chinese Spring carrying both arms of chromosome 7D as telosomes. The seeds were kindly provided by Prof. B.S. Gill (KSU, Manhattan, KS) and Prof. A. Lukaszewski (UC, Riverside, CA). Liquid suspensions of intact chromosomes were prepared according to Kubaláková *et al*. (2002) by mechanical homogenization of 20–25 formaldehyde‐fixed root‐tip meristems enriched for metaphase cells in 1 mL ice‐cold isolation buffer (IB; Šimková *et al*., 2003). Chromosomes in suspension were stained with 2 μg/mL DAPI (4′,6‐diamidino‐2‐phenylindole) and analysed using a FACSAria SORP flow cytometer (Becton Dickinson, San Jose, CA). Purity of the flow‐sorted 7DS arm as estimated by FISH was 84%. The major contaminant in the sorted fraction was the 7DL telosome, which formed 1.1% of the sorted fraction; the remaining ~15% were made up of a mixture of other chromosomes. In total, 1.6 × 10^6^ 7DS arms corresponding to 1.2 μg DNA were flow‐sorted and embedded in three agarose miniplugs of total volume 60 μL. DNA embedded in plugs was purified by proteinase K (Roche) treatment as described in Šimková *et al*. (2003). The miniplugs were washed four times in wash buffer (10 mm Tris, 50 mm EDTA, pH 8.0) and four times in TE buffer (10 mm Tris, 1 mm EDTA, pH 8.0), melted for 2 min at 70 °C and solubilized with GELase (Epicentre, Madison, CA) for 45 min. The purified DNA underwent 30 min of drop dialysis (Merck Millipore, Billerica, MA) against TE buffer and was quantified using Quant‐iT^™^ PicoGreen^®^ dsDNA assay (Thermo Fisher Scientific, Waltham, MA).

Survey sequences of the 7DS chromosome arm (Berkman *et al*., 2011; International Wheat Genome Sequencing Consortium (IWGSC), 2014) were inspected for frequency of recognition sites of particular nicking enzymes, and nicking endonuclease *Nt.BspQI* with an estimated frequency of 12 sites/100 kb (labelling frequency) was selected for the labelling. DNA was labelled using the IrysPrep^®^ Reagent Kit (BioNano Genomics, San Diego, CA) following manufacturer's instructions with modifications suitable for samples with lower DNA concentration. Specifically, 200 ng of purified chromosomal DNA were nicked using 2U of *Nt.BspQI* (New England BioLabs, Beverly, MA) at 37 °C for two hours in NEBuffer 3. The nicked DNA was labelled with a fluorescent‐dUTP nucleotide analogue using Taq polymerase (New England BioLabs) for one hour at 72 °C. After labelling, the nicks were ligated with Taq ligase (New England BioLabs) in the presence of dNTPs. The backbone of the labelled DNA was stained with IrysPrep^®^ DNA Stain (BioNano Genomics).

Labelled and stained DNA was loaded on the Irys chip and run for two runs for a total of 41 cycles. A total of 82.5 Gb data were generated, of which 68.8 Gb exceeded 150 kb. After single molecules were detected to find the label positions on the DNA backbone, *de novo* assembly was performed by a pairwise comparison of all single molecules and graph building (Cao *et al*., 2014). A *P*‐value threshold of 10e^−9^ was used during the pairwise assembly, 10e^−10^ for extension and refinement steps, and 10e^−11^ for a final refinement.

### Repeat detection and analysis

An algorithm included in the IrysView 2.0 software package (BioNano Genomics) was used to identify tandem repeats with one nick site per repeat motif (labelled tandem repeats), in both the assembly and the raw data. Detected repeats were quantified and their unit size and frequency in the dataset were plotted in a histogram for visual analysis. Arrays of five or more repeat units were considered in our analysis.

### Physical map construction, anchoring and sequencing

The 7DS physical contig map (https://urgi.versailles.inra.fr/gb2/gbrowse/wheat_phys_7DS_v1/) was constructed from a 7DS‐specific BAC library using FPC software (Soderlund *et al*., 2000) as described in Šimková *et al*. (2011). A MTP of 4608 BAC clones selected from the physical map were sequenced using a pooling strategy in which 96 pools, each consisting of four BACs, were indexed and sequenced on a single lane using the Illumina HiSeq 2000 platform. Sequences were de‐multiplexed and assembled using the SASSY assembler (Kazakoff *et al*., 2012). Deconvolution was supported by BAC‐end sequences generated from the MTP BAC clones by Sanger sequencing. The resulting assembly has a mean N50 of 65 kb and currently covers about 75% of the 7DS arm. The sequence contigs were used for *in silico* anchoring of the 7DS physical map to the *Ae. tauschii* SNP genetic map (Luo *et al*., 2013) and a consensus bread wheat DArTseq genetic map (A. Kilian, unpublished). 7DS‐specific microsatellite markers from GrainGenes database (http://wheat.pw.usda.gov/GG2/index.shtml) were anchored manually by PCR screening of three‐dimensional pools of the 7DS BAC library (Šimková *et al*., 2011).

### Anchoring 7DS sequence assemblies to the genome map

Comparison of sequence assembly with the genome map was performed using the IrysView 2.0 software package. Based on the type of analysis, individual sequences representing 7DS MTP clones or complete pools of MTP BAC clones were compared with the complete set of genome maps or with individual genome maps, respectively. Prior to comparison, *cmap* files were generated from *fasta* files of individual sequences or BAC pools, respectively. Query‐to‐anchor comparison was performed with default parameters and variable *P*‐value threshold ranging from 1e^−6^ to 1e^−10^, based on the type of analysis.

To estimate the minimum length of sequence needed for identifying the corresponding genome map, we randomly selected ten MTP BAC clones with inserts exceeding 120 kb assembled as a contiguous sequence (Table S1). BAC clone sequences, checked for the typical frequency of nicking sites (~12 sites/100 kb), were compared with the whole set of genome maps as described above. Clone assignments to particular genome maps were validated by anchoring overlapping or neighbouring clones identified previously by FPC. Subsequently, sequences of the analysed BAC clones were truncated to the length of 120 kb and analysed using a sliding window approach, applying three window sizes ‐ 30, 60 and 90 kb ‐ and a window shift of 10 kb. All generated sequence fragments were compared with the complete set of genome maps in IrysView software, which calculated a confidence value for each of the aligned sequences as –log 10 (*P*‐value) where the p‐value calculation is described in Anantharaman and Mishra (2001). To maximize the number of aligned sequences, the *P*‐value threshold was set to 10e^−4^.

## Conflict of interest

Alex R. Hastie and Saki Chan are employees of BioNano Genomics.

## Supporting information


**Figure S1** Molecule size distribution obtained by analysing 7DS HMW DNA on the Irys chip.
**Figure S2** Quantitation of labelled tandem repeats in the complete set of raw data >150 kb obtained for the 7DS arm. Arrays of minimum 5 units were considered. (a) Scale 0.6 kb, (b) scale 0.1 kb.

**Table S1** BAC clones used for the sliding window analysis.
**Table S2** BAC contigs, MTP clones and markers anchored to genome map No. 19.
