# Supplementary material for: BioNano genome mapping of individual chromosomes supports physical mapping and sequence assembly in complex plant genomes
Source: Plant Biotechnol J. 2016 Jan 23;14(7):1523–31. doi: 10.1111/pbi.12513 (PMC5066648; doi:10.1111/pbi.12513)
Supplement: Supplementary file 1 — Figure S1 Molecule size distribution obtained by analysing 7DS HMW DNA on the Irys chip. Figure S2 Quantitation of labelled tandem repeats in the complete set of raw data >150 kb obtained for the 7DS arm. Arrays of minimum 5 units were considered. (a) Scale 0.6 kb, (b) scale 0.1 kb. [file PBI-14-1523-s001.docx]

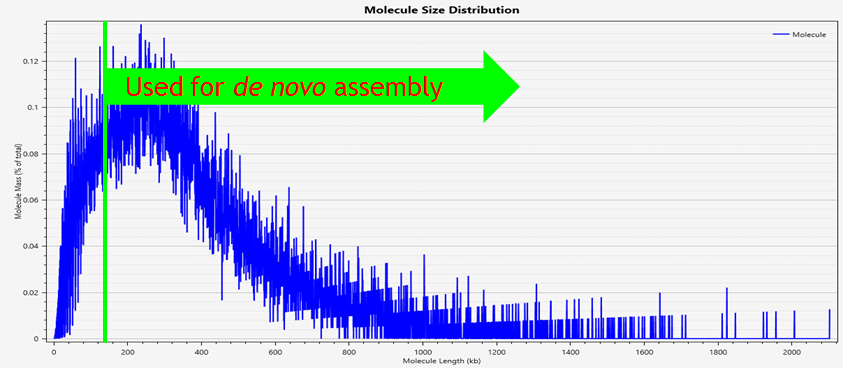


**Suppl. Figure 1. Molecule size distribution obtained by analyzing 7DS HMW DNA on the Irys chip**


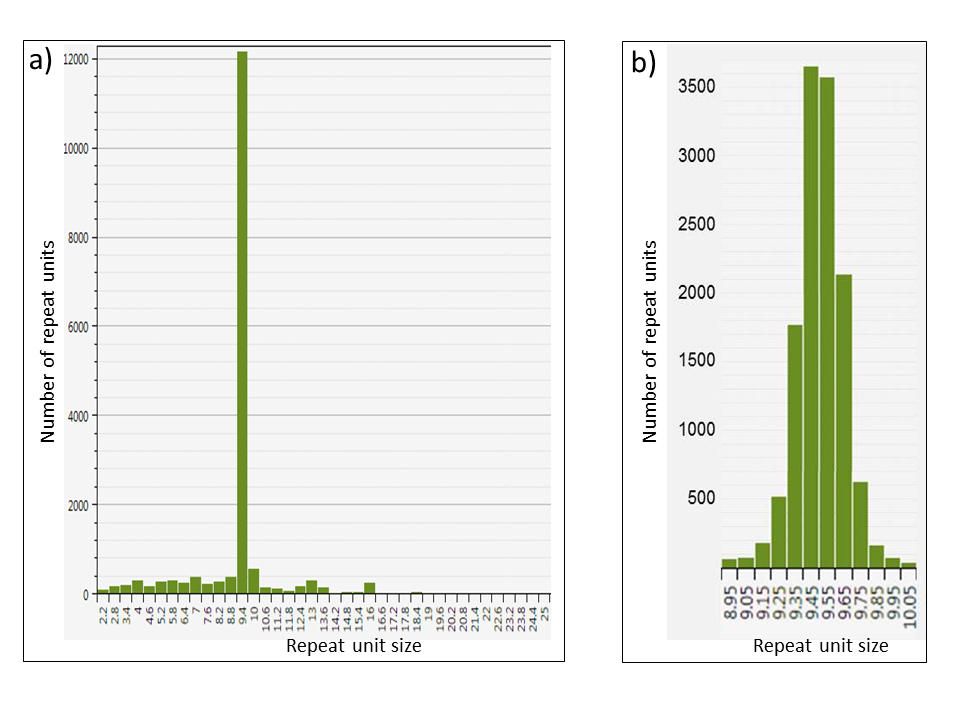


**Suppl. Figure 2. Quantitation of labelled tandem repeats in the complete set of raw data >150 kb obtained for the 7DS arm.** Arrays of minimum 5 units were considered. (a) Scale 0.6 kb, (b) scale 0.1 kb.
