# Supplementary material for: BioNano genome mapping of individual chromosomes supports physical mapping and sequence assembly in complex plant genomes
Source: Plant Biotechnol J. 2016 Jan 23;14(7):1523–31. doi: 10.1111/pbi.12513 (PMC5066648; doi:10.1111/pbi.12513)
Supplement: Supplementary file 2 — Table S1 BAC clones used for the sliding window analysis. Table S2 BAC contigs, MTP clones and markers anchored to genome map No. 19. [file PBI-14-1523-s002.docx]

**Suppl. Table 1. BAC clones used for the sliding window analysis**

| **BAC clone** | **Length (bp)** | **Genome map No.** | **Confidence**  **complete BAC** | **Confidence**  **120-kb sequence** |
| --- | --- | --- | --- | --- |
| TaaCsp7DS081C04 | 147,078 | 62 | 19.01 | 14.51 |
| TaaCsp7DS082B16 | 128,143 | 186 | 24.89 | 24.89 |
| TaaCsp7DS082J18 | 126,013 | 30 | 21.70 | 21.70 |
| TaaCsp7DS082M03 | 143,891 | 140 | 18.83 | 13.62 |
| TaaCsp7DS084L01 | 121,365 | 49 | 20.91 | 20.91 |
| TaaCsp7DS103L24 | 136,697 | 12 | 22.12 | 19.19 |
| TaaCsp7DS115G05 | 150,313 | 56 | 15.85 | 12.88 |
| TaaCsp7DS116C21 | 159,744 | 20 | 17.46 | 14.22 |
| TaaCsp7DS117B16 | 128,495 | 93 | 21.36 | 17.83 |
| TaaCsp7DS121A05 | 134,356 | 42 | 21.50 | 18.84 |

**Suppl. Table 2. BAC contigs, MTP clones and markers anchored to genome map No. 19**

| **7DS ctg** | **MTP BAC clones** | **Marker** |
| --- | --- | --- |
| **ctg454** | TaaCsp7DS102J17 |  |
|  | TaaCsp7DS110I15 |  |
|  | TaaCsp7DS063L03 |  |
|  | TaaCsp7DS002N16 |  |
|  | TaaCsp7DS098F02 |  |
| **ctg962** | TaaCsp7DS120O16 |  |
|  | TaaCsp7DS125A07 |  |
|  | TaaCsp7DS030E09 | AT7D6224 |
|  | TaaCsp7DS090C07 |  |
|  | TaaCsp7DS089D19 |  |
|  | TaaCsp7DS073L11 | AT7D6223 |
| **ctg713** | TaaCsp7DS004A06 |  |
|  | TaaCsp7DS080K03 |  |
|  | TaaCsp7DS080J09 |  |
|  | TaaCsp7DS114G21 |  |
|  | TaaCsp7DS037J06 |  |
|  | TaaCsp7DS021L16 |  |
| **ctg763** | TaaCsp7DS114C15 |  |
|  | TaaCsp7DS101G14 |  |
| **ctg546** | TaaCsp7DS010P24 | AT7D6220 |
|  | TaaCsp7DS057N22 |  |
|  | TaaCsp7DS074K06 | AT7D6219 |
|  |  | AT7D6218 |
|  | TaaCsp7DS004P16 |  |
|  | TaaCsp7DS026C11 |  |
|  | TaaCsp7DS042F15 |  |
|  | TaaCsp7DS049G07 |  |
|  | TaaCsp7DS045O06 |  |
|  | TaaCsp7DS115E18 |  |
|  | TaaCsp7DS101A11 |  |
|  | TaaCsp7DS072N09 |  |
| **ctg1080** | TaaCsp7DS035I07 | AT7D6216 |
|  | TaaCsp7DS119E15 |  |
|  | TaaCsp7DS119E15 | AT7D6217 AT7D6210 |
|  | TaaCsp7DS101A23 |  |
|  | TaaCsp7DS023M12 |  |
| **ctg3912** | TaaCsp7DS089E21 |  |
|  | TaaCsp7DS056L11 |  |
|  | TaaCsp7DS075H08 |  |
|  | TaaCsp7DS110G13 |  |
| **ctg3865** | TaaCsp7DS110C03 |  |
|  | TaaCsp7DS128G06 |  |
|  | TaaCsp7DS108G01 |  |
|  | TaaCsp7DS050G16 | AT7D6209 |
|  | TaaCsp7DS067A19 |  |
| **ctg1857** | TaaCsp7DS007P19 | AT7D6214 |
|  | TaaCsp7DS036F21 |  |

Red colour indicates the incorrectly assigned clone.
